# Calcium Channel Blockers as Tocolytics: Principles of Their Actions, Adverse Effects and Therapeutic Combinations

**DOI:** 10.3390/ph6060689

**Published:** 2013-05-23

**Authors:** Róbert Gáspár, Judit Hajagos-Tóth

**Affiliations:** Department of Pharmacodynamics and Biopharmacy, Faculty of Pharmacy, University of Szeged, H-6701, P.O. Box 121, Hungary; E-Mail: judittoth@pharm.u-szeged.hu

**Keywords:** nifedipine, preterm labour, tocolysis, combination

## Abstract

Dihydropyridine Ca^2+^ channel blockers (CCBs) are widely accepted in the treatment of premature labour. Their mechanism of action in tocolysis involves the blockade of L-type Ca^2+^ channels, influenced by the Ca^2+^-activated K^+^ channels, beta-adrenergic receptors (β-ARs) and sexual hormones. In clinical practice, most experience has been gained with the use of nifedipine, whose efficacy is superior or comparable to those of β-agonists and oxytocin antagonists. Additionally, it has a favourable adverse effect profile as compared with the majority of other tocolytics. The most frequent and well-tolerated side-effects of CCBs are tachycardia, headache and hypotension. In tocolytic therapy efforts are currently being made to find combinations of tocolytic agents that yield better therapeutic action. The available human and animal studies suggest that the combination of CCBs with β-AR agonists is beneficial, although such combinations can pose risk of pulmonary oedema in multiple pregnancies and maternal cardiovascular diseases. Preclinical data indicate the potential benefit of combinations of CCBs and oxytocin antagonists. However, the combinations of CCBs with progesterone or cyclooxygenase inhibitors may decrease their efficacy. The CCBs are likely to remain one of the most important groups of drugs for the rapid inhibition of premature uterine contractions. Their significance may be magnified by further clinical studies on their combined use for tocolysis.

## 1. Introduction

Pre-term birth (PTB) is one of the main clinical problems in obstetrical practice. The incidence of PTB may differ in different regions, the rate varying worldwide in the range between 5–11% [1]. Despite of the availability of several drugs that inhibit pre-term contractions (tocolytics), the pharmacotherapy of PTB is inappropriate. Additionally, the maternal and foetal side-effects caused by high doses of such drugs may induce further complications; there is therefore a great need for effective and well-tolerated drugs against PTB. Ca^2+^ channel blockers (CCBs), and especially nifedipine and nicardipine, are among the frequently used tocolytics. They are constantly gaining in importance, and increasingly more significant than traditional tocolytics such as beta-adrenergic receptor (β-AR) blockers or magnesium sulphate. Although they do not meet the criteria of ideal tocolytics, CCBs have certain properties that make them preferable to other tocolytics. Haas *et al*. concluded that CCBs have high probability of delaying PTB and improving neonatal outcomes [2].

## 2. The Mechanism of Action of CCBs on the Pregnant Myometrium

Uterine contractile activity is regulated by the increase in intracellular Ca^2+^ concentration in the myometrial cells. Voltage-gated Ca^2+^ channels (VGCCs) mediate the Ca^2+^ influx in response to membrane depolarization and regulate intracellular processes such as contraction [3,4]. Ca^2+^ binds to calmodulin and activates the myosin light chain kinase (MLCK) in the myometrial cells and therefore leads to the phosphorylation of serine 19 on myosin light chains and initiates subsequent cross-bridge cycling. There are two sources for the increase in activator Ca^2+^: entry across the surface membrane through VGCCs and/or release from the sarcoplasmic reticulum. In the uterus, where the action potential occurs, the resulting depolarization and consequent opening of the VGCCs make this the major source of Ca^2+^ for contraction. Each contraction is accompanied by a Ca^2+^ transient in the uterus, and both transients and contractions are abolished if the VGCCs are blocked [5,6,7].

The voltage dependent L-type calcium channels have been identified in uterine myometrium by electrophysiological, pharmacological and molecular studies [8]. They are responsible for the majority of the observed calcium current in the human myometrium. The Ca^2+^ channels are complex proteins composed of five distinct subunits (α_1_, α_2_, β, δ and γ) encoded by multiple genes [3]. Dihydropyridines (DHPs) such as nifedipine bind to the DHP binding side of the VG L-type channels, which is located on the α1 subunit. The channels have several isoforms resulting from alternative splicing sites of the α1 subunit [9,10]. The increased expression of one of the VGCC isoforms (S3B) was detected in pregnant rat myometrium during labour [11]. In guinea pigs, Collins *et al.* [8] have demonstrated significant changes in the expression of the α1 subunit of L-type VGCCs in pregnancy and labour. An increase in DHP binding capacity was seen through the last half of gestation which supports the role of L-type VGCCs in the process of parturition.

The CCBs therefore arouse considerable interest for both therapeutic and experimental purposes [12]. The activity and sensitivity of L-type Ca^2+^ channels to DHP CCBs are influenced by at least three factors.

### 2.1. Factor 1: Ca^2+^-Activated K^+^ (BK_Ca_) Channels

The uterus contains BK_Ca_ channels and their expression and distribution have been shown to be gestation-regulated [13]. The BK_Ca_ channels are a diverse group of K^+^ channels that participate in the repolarization and hyperpolarization of action potentials. They are activated by elevated intracellular Ca^2+^ levels. The opening of BK_Ca_ channels is associated with small hyperpolarizations, which lead to the decreased opening of L-type Ca^2+^ channels and a fall in Ca^2+^ concentration, and hence to relaxation [5,14,15]. The role of BK_Ca_ channels in combination with nifedipine was investigated by Moynihan *et al.* [16], who concluded that the BK_Ca_ channel blockers significantly antagonize the relaxant effect of nifedipine. However, another study, with paxilline and tetraethylammonium, indicated that the BK_Ca_ channels and any other K^+^ channel, in contrast with human myometrium, are not involved in the relaxing effect of nifedipine in the pregnant rat myometrium [17].

### 2.2. Factor 2: Beta-Adrenergic Receptors

The adrenergic system plays an important role in the control of uterine contractility. Currently, β_2_-AR agonists are still among the most frequently used tocolytics, although their therapeutic significance in PTB is constantly questioned.

β-AR stimulants are known to produce smooth muscle cell relaxation by activating Gs proteins, and their Gαs subunit stimulates adenyl cyclase. This elevates the level of cyclic adenosine monophosphate (cAMP), which activates protein kinase A, this activated form inducing phosphorylation of the Ca^2+^ channels. This mechanism is well known in the heart muscle [18] and may be similar to that in the pregnant myometrium. One of the first studies of the effects of combinations of β_2_-agonists and CCBs was reported by Lever *et al.* [19]. The literature data show that both isradipine and nifedipine potentiate the relaxant action of terbutaline and salmeterol in the isolated trachea [20]. Together, these results suggest an increased relaxant effect of β_2_-agonists combined with CCBs in the pregnant myometrium. The efficacy of a β_2_-AR agonist and a CCB in the pregnant myometrium has been investigated both *in vitro* and *in vivo*. Synergism has been observed in the uterus-relaxing effect of nifedipine and the β_2_-AR agonist terbutaline, although the extent of potentiation depends on the sequence of administration of the two compounds. Terbutaline possibly activates the Ca_v_1.2 channels and decreases the maximum relaxing effect of nifedipine. The resultant effect of the increase in cAMP level and the activation of Ca_v_1.2 channels cause a weaker smooth muscle relaxation. In the opposite case, when nifedipine is administered first, the Ca_v_1.2 channels are blocked, hence terbutaline cannot activate them [21].

### 2.3. Factor 3: Progesterone

Another factor which regulates the L-type Ca^2+^ channel is the progesterone (P4)/oestrogen (E2) ratio [22]. P4 is a key component in the complex regulation of the normal female reproductive function. It plays a central role in the maintenance of pregnancy and in the initiation of parturition by modulating myometrial contractility and excitability. P4 supports pregnancy and prevents parturition by promoting myometrial quiescence [23,24]. The level of P4 normally declines at term prior to the development of labour, and P4 has therefore also been used as prophylaxis in the prevention of PTB [25,26,27,28].

Combinations of P4 and β_2_-AR agonists have also been investigated in the pregnant myometrium. P4 pre-treatment increases the expression of the β_2_-AR during pregnancy and alters the effects of β_2_-AR agonists on the pregnant myometrium; P4 and its derivatives have been considered as drugs against PTB [23,29].

It was been demonstrated that the mRNA expression of the pore-forming α_1C_ subunit of the L-type Ca^2+^ channel is regulated by glucocorticoid hormones, but tissue-specific changes may occur [30]. Biochemical experiments have detected the presence of two forms of the L-type Ca^2+^ channel in native tissues: a short form (α_1C-short_) and a long (α_1C-long_) form. Helguera *et al*. [22] established that a P4-mediated mechanism favours the expression of the long form, in the presence of which the channel displays lower activity. We earlier observed [21] that *in vivo* P4 pre-treatment (for 7 days) decreased the maximum inhibitory effect of nifedipine and increased its EC_50_* in vitro*, and also abolished the ability of nifedipine to delay labour in hormone-induced pre-term delivery in rats *in vivo* [31]. These results correlate with the hypothesis that P4 decreases the activity of the L-type Ca^2+^ channels. However, Baumbach *et al*. [32] reported that P4 increased the relaxing effect of nifedipine on the human myometrium *in vitro*. In that study, P4 was added directly to the tissue bath. It is known that the prompt action of P4 on the contractility of the smooth muscles is not caused by genomic effects through a receptor-mediated mechanism, but by direct effects on the plasma membrane [33]. The manners in which the three factors influence the L-type Ca^2+^ channels mentioned above are presented in Table 1.

**Table 1:** Influence of some physiological factors on L-type Ca^2+^ channels in the pregnant myometrium.

Physiological factor	Effect on L-type Ca^2+^ channel	Possible clinical relevance
BK_Ca_ channel activation	inhibition	presumably increases the tocolytic effect of CCBs
β-AR activation	opening	increases the tocolytic effect of CCBs
Long-term progesterone effect	expression of less sensitive form	presumably decreases the tocolytic effect of CCBs

## 3. The Efficacy of CCBs against PTB

A number of drugs are used against threatening PTB, although only oxytocin antagonists have been designed specifically as tocolytic medication (Table 2). Thus, DHP CCBs are utilized for this purpose, but they have not been licensed for tocolysis. Many data demonstrate the efficacy of nifedipine, whereas little information is available on the action of nicardipine in PTB.

Nifedipine is used orally for both tocolysis and cardiovascular treatment. The appropriate dose of nifedipine for tocolysis is still under investigation. In a comparison of two dose regimens of oral nifedipine in a study on threatening PTB between gestational weeks 24 and 34, it was found that a high dose of the drug (20 mg loading dose, daily 120–160 mg for 48 hours, followed by 80–120 mg daily up to 36 weeks) did not have any advantage over a low dose (10 mg loading dose, daily 60–80 mg for 48 hours, followed by 60 mg daily up to 36 weeks) as regards uterine quiescence [34]. In a small Bulgarian study, nifedipine (40 mg four times daily) delayed a large majority of the pregnancies (32 out of 41) with early contractions until normal term, without significant side-effects [35].

**Table 2:** Class of drugs used clinically for tocolysis.

Class of drugs	Primarily licensed for	The most frequent side effects
Beta-adrenergic agonists	bronchial asthma	Maternal: tachycardia, hyperglycemia, pulmonary oedema Foetal: tachycardia, RDS
Cyclooxygenase inhibitors	inflammation, pain, fever	Foetal: premature closure of ductus arteriosus, reduced amniotic fluid index
Gestagens	hormonal substitution, contraception	no relevant side effect
Magnesium sulphate	hypomagnesaemia, eclampsia in pregnancy	Maternal: constipation, visual blurring, headache
CCBs (DHPs)	hypertension	Maternal: headache, tachycardia, hypotension
Oxytocin antagonists	tocolysis	Maternal: tachycardia, chest pain

There have been reports of the lack of action of nifedipine in premature contractions. A placebo-controlled, randomized trial demonstrated that 20 mg of nifedipine administered every 4–6 hours did not maintain pregnancy or delay delivery as compared with the placebo group [36]. In a recent trial, nifedipine-maintained tocolysis (80 mg/day orally for 12 days) did not result in a statistically significant reduction in adverse perinatal outcome or in a significantly lengthened gestational duration in patients with threatening PTB, although the rate of adverse perinatal outcome was lower as compared with the control group [37]. This result suggests that the use of nifedipine does not significantly delay labour.

In twin pregnancies where nifedipine tocolysis was administered a switchover to subcutaneous terbutaline following hospitalization for recurrent symptoms of the threatening PTB had a positive impact on pregnancy prolongation and on the neonatal outcome [38].

In another investigation, nifedipine was found to be more effective than indomethacin in inhibiting uterine contractions during the ﬁrst 2 hours; however, there was no difference between indomethacin and nifedipine in delaying delivery for up to 7 days [39].

Atosiban and nifedipine have been shown not to significantly differ in delaying delivery [40,41]. This limited evidence suggests no essential differences in the tocolytic efficacy of these two drugs. The oral administration, the lower costs and the possibly lower level of neonatal morbidity favour the use of nifedipine [42,43].

Nicardipine has the advantageous feature over nifedipine that its intravenous administration is possible, and it is the first choice for some obstetricians in the management of PTB. However, intravenous nicardipine does not increase the duration of pregnancy in comparison with oral nifedipine. The median duration between treatment for PTB and delivery was significantly longer when nifedipine was used [44]. Oral nicardipine is additionally an effective and well-tolerated tocolytic agent. It is able to arrest PTB more rapidly than parenteral magnesium sulphate [45].

Nicardipine was earlier found to be as effective as salbutamol in the treatment of PTB, and it was suggested to have advantages especially in cases of pregnancy accompanied by hypertension, diabetes or maternal cardiopathy [46]. A comparison of nicardipine with salbutamol in a small Tunisian study revealed no significant difference in the efficacies of the two compounds as concerns the average time for the disappearance of uterine contractions. However, fewer secondary adverse effects were detected with nicardipine, which was therefore proposed as the tocolytic of first choice [47].

## 4. Side-Effects of CCBs during Tocolysis

CCBs are generally well tolerated. The most frequent maternal side-effect is headache which is associated with the transient hypotension caused by the loading doses [48]. Maternal tachycardia is the second most frequently reported adverse events following nifedipine therapy. Besides maternal headache, anxiety and vomiting are other frequent side-effects described for nifedipine [49]. As minor side-effects, nifedipine may cause palpitation and flushing during tocolysis, but these occur in fewer than 10% of the patients [50]. Nifedipine does not influence foetal movement, heart rate or blood flow and thus appears to have no direct adverse foetal effects [51]. It is known that the side-effects of the drug do not correlate with its plasma level. The adverse reactions can occur in a wide range of nifedipine plasma concentrations (6–101 ng/mL), and therefore there is no need to adjust the dose of nifedipine to body weight, body mass index or gestational age [52].

Nifedipine was suspected of being responsible for severe dyspnoea in seven patients with multiple pregnancies. It was hypothesized that the complications were due to the imbalance in the ventilation/perfusion of the lungs in consequence of the diaphragm being elevated due to the pregnancy. These changes are usually more pronounced in twin pregnancies. The administration of nifedipine resulted in a ventilation-perfusion discrepancy and dyspnoea. These observations question the safety of nifedipine as a tocolytic agent in patients with multiple pregnancies [53]. Additionally, tocolysis with oral nifedipine has been reported to be responsible for maternal pulmonary oedema [54].

A comparison of the side-effects of nifedipine and nicardipine revealed that the patients in the nicardipine-treated group had significantly more side-effects (31% *versus* 16% for the nifedipine group), although the hypotensive effect of nifedipine was higher [55].

Maternal pulmonary oedema induced by the infusion of nicardipine was reported in five pregnant women during tocolysis. Therapy was discontinued immediately after the diagnosis, but two patients required admission to the intensive care unit [56]. Three other cases of maternal pulmonary oedema during PTB were associated with the combination of salbutamol and intravenous nicardipine. It was recommended that the association of CCBS and beta-agonists for the treatment of PTB should be avoided [57].

## 5. Tocolytic Effect of CCBs in Combination with Other Drugs

The idea of combined tocolytic therapy with nifedipine is not new. The first report on effective tocolysis with a combination of nifedipine and beta-mimetic terbutaline was published in 1985 [58]. The effect was dramatic; the duration of pregnancy was delayed for 2 months without any significant side-effects. Unfortunately, this successful treatment was carried out on only one patient. The efficacy of the combination of nifedipine and salmeterol was also proved in a hormonally-induced PTB model involving rat and human myometrial strips from caesarean sections in term pregnancies [29]. These promising results were overshadowed by findings of clinical disadvantages of the combination of CCBs and β-mimetics, e.g., possible increases in the risk of maternal myocardial infarction and pulmonary oedema. As mentioned earlier, the CCBs are able to elicit respiratory problems in monotherapy, and this property is retained when they are applied in combination with other drugs, such as glucocorticoids and atosiban, especially in multiple pregnancies [59]. Interestingly, a recent study has revealed that atosiban alone is able to induce dyspnoea and pulmonary oedema in multifoetal pregnancies [60]. Although some authors suggest that the combination of CCBs and β-mimetics should be avoided, this may be restricted to twin pregnancies and maternal cardiovascular disorders.

One study has been carried out to investigate the efficacy of combinations of CCBs and oxytocin antagonists for tocolysis. The combination atosiban + nifedipine exerts on additive tocolytic effect on the contractility of myometrial strips in both pre-term and term patients. Surprisingly, the combination nifedipine + celecoxib exhibited a weaker effect than that of nifedipine alone [61]. The combination of nifedipine and atosiban may therefore merit testing in clinical studies.

P4 analogues have been re-introduced into tocolytic therapy in the past decade as preventive compounds against PTB. For women with a history of spontaneous PTB, P4 reduces the incidence of PTB before 34 weeks, but has no beneficial effect in multiple pregnancies or in the event of a short cervix [62].

P4 pretreatment has been found to alter the structure of Ca^2+^ channels, promoting the expression of the less sensitive form of the channel, and to weaken the tocolytic effect of nifedipine in pregnant rats [20,29]. The tocolytic effect of the combination of 17-α-hydroxyprogesterone caproate and nifedipine was not superior to that of nifedipine alone in a randomized clinical trial [63]. This result suggests that P4 treatment probably does not worsen the tocolytic efficacy of CCBs in humans. The modification of the tocolytic effect of nifedipine by other compounds and the potential risks of the combinations are indicated in Table 3.

**Table 3:** Myometrial effects of different combinations of nifedipine.

Combination	Change in myometrial relaxation	Risk of combination
Nifedipine + ritodrine	increased (in both human and animal studies *in vivo*)	pulmonary oedema, myocardial infarction
Nifedipine + atosiban	increased (in a human study *in vitro*)	no information (data from *in vitro* experiments only)
Nifedipine + celecoxib	decreased (in a human study *in vitro*)	no information (data from *in vitro* experiments only)
Nifedipine + progesterone	decreased (in an animal study *in vivo*) unchanged (in a human study *in vivo*)	no special risk as compared with nifedipine monotherapy

## 6. Conclusions

CCBs are increasingly used for tocolysis, although officially they are not licensed for this purpose. Their efficacy is at least equivalent to that of the traditionally used drugs, but their adverse effects seem to be milder and better tolerated than those of beta-adrenergic agonists and not more severe than those of oxytocin antagonists. Unfortunately, the CCBs in monotherapy are not better than other tocolytics in long-term tocolysis (pregnancy maintenance).

The effects of nifedipine in tocolytic therapy may possibly be intensified through their combination with other compounds. Their efficacy may be enhanced by low concentrations of β_2_-mimetics. However, the administration of β_2_-adrenergic agonists cannot precede that of nifedipine. There are a number of clinical findings that warn against the combination of these two groups in cases of multiple pregnancies and maternal cardiovascular disorders. On the other hand, their combination may be investigated in single pregnancies that require tocolysis. With regard to the limited nature of the information available from preclinical studies, a trial relating to the combined tocolytic effect of CCBs and atosiban may be reasonable.

Overall, it may be concluded that DHP CCBs should remain one of the most important group of drugs for the rapid inhibition of premature uterine contractions. There is an urgent need for an updated systematic review (e.g., according to the principles of Cochrane reviews) on the clinical application of CCBs in premature labour. Additionally, further clinical studies are required to investigate how to enhance their efficacy in combination with other drugs.
